# Loneliness in Bereavement: Measurement Matters

**DOI:** 10.3389/fpsyg.2021.741762

**Published:** 2021-09-13

**Authors:** Anneke Vedder, Margeret S. Stroebe, Henk A.W. Schut, Kathrin Boerner, Jeffrey E. Stokes, Paul A. Boelen

**Affiliations:** ^1^Department of Clinical Psychology, Utrecht University, Utrecht, Netherlands; ^2^Department of Clinical Psychology & Experimental Psychopathology, University of Groningen, Groningen, Netherlands; ^3^Department of Gerontology, University of Massachusetts Boston, Boston, MA, United States; ^4^ARQ National Psychotrauma Centre, Diemen, Netherlands

**Keywords:** loneliness, measurement, assessment, social isolation, bereavement, grief, prolonged grief disorder, complicated grief

## Abstract

The role of loneliness in the bereavement experience has been reported as substantial, with the death of a close person leaving a considerable void in the life of the bereaved. Yet, there is lack of agreement about its precise role and, notably, whether loneliness should be included as a core symptom for diagnosis of grief complications. The ongoing threat of heightened social isolation due to the COVID-19 pandemic underlines the need to understand the impact of loneliness, and to accurately chart its prevalence, intensity, duration, and associated difficulties in the context of bereavement. Assessment issues are central to this endeavor. In this article, we review the scientific literature to examine how loneliness after bereavement has been operationalized and measured. Sixty-three articles analyzing 51 independent datasets were reviewed. Results show major disparities: approximately half of the projects assessed loneliness by means of one of two validated scales (spanning different versions); the remainder included only single- or few-item measures. Diverse instructions, content and answer categories were used. While one size does not fit all, awareness of assessment options and dis/advantages may aid selection of the most appropriate measure, to suit the goals of a particular study and the specific groups under investigation. Our conclusion is that, in selecting a loneliness measure, health care professionals should come to their own well-informed decision, aided by the information provided in our review.

## Introduction

Loneliness is generally understood to be a distressing reaction accompanying the perception that one's social needs are not being met by one's interpersonal relationships (Hawkley and Cacioppo, 2010). It has been established as a risk factor for compromised health and well-being, not only in terms of general mental and physical illness, morbidity, and mortality, but also for specific problems such as reduced daily functioning and suicidal ideation, risky health behaviors, age-related disease such as Alzheimer's, and various physiological indices of ill-health (Hawkley and Cacioppo, 2003; Leigh-Hunt et al., 2017; Brown et al., 2018; Wang et al., 2018). A range of theoretical approaches has been offered in the scientific literature to help explain such phenomena and manifestations of loneliness, with major contributions including the classic attachment theory extension by Weiss (1973); the cognitive discrepancy approach of Perlman and Peplau (1981) and the evolutionary perspective of Cacioppo et al. (2014). For an extended review of these theories, see De Jong-Gierveld et al. (2018) and Marangoni and Ickes (1989).

With respect to bereavement, research has confirmed that loneliness is frequently one of the major challenges experienced following loss (Vedder et al., 2021). Particular research attention has been paid to its impact on older persons who have lost their partner (Lund, 1989; Utz et al., 2014). There are indications that loneliness may play a key role in depression after the loss of a close person, as well, functioning as a key symptom that may lead from bereavement to the development of other listed depressive symptoms (Fried et al., 2015). Loneliness is also associated with complications in grieving, including post-traumatic stress disorder, depression, as well as other mental and physical health problems (e.g., Simon et al., 2014; Erzen and Çikrikci, 2018; Asch et al., 2021).

A recent review of empirical studies of loneliness in bereavement testifies to the greater frequency and/or intensity of loneliness among bereaved compared with non-bereaved groups (Vedder et al., 2021). Yet, loneliness does not appear consistently in lists of core symptoms in diagnostic manuals of mental disorders. Different sets of criteria for disturbed grief have been proposed (Boelen and Lenferink, 2020). In the forthcoming text revision of the 5th edition of the Diagnostic and Statistical Manual for Psychiatric Disorders (DSM-5-TR; American Psychiatric Association and Association, 2013; Prigerson et al., 2021), “Intense loneliness (i.e., feeling alone or detached from others) as a result of the death” is listed as a symptom among the criteria for Prolonged Grief Disorder (PGD). Similarly, “Loneliness” is one of the four separation distress symptoms of Complicated Grief (CG) proposed by Shear et al. (2011). By contrast, there is no symptom of loneliness among those for PGD as described in the 11th edition of the International Classification of Diseases (ICD-11; World Health Organization, 2019).

One plausible reason for the discrepancy in recognition of loneliness as a key feature of complications in grief is that there is still quite limited knowledge regarding the nature, prevalence, and intensity of loneliness and loneliness-related problems encountered by bereaved people. In our view, matters relating to assessment contribute to this shortage of information. Examination of the general (i.e., non-bereavement-specific) loneliness literature points to a lack of agreement on how best to measure loneliness or what precisely such assessment(s) should incorporate (for example, whether to measure a single or multiple related constructs; Cramer and Barry, 1999). This likely also reflects the difficulties associated with capturing the multidimensionality and complexity of the construct (Yanguas et al., 2018). Nevertheless, providing a useful starting point for further consideration, there is reasonable consensus regarding a general definition of loneliness (for a review of definitions, see: De Jong Gierveld, 1998; Hawkley and Cacioppo, 2010). According to Valtorta and Hanratty (2012), “*One of the most widely-used definitions has loneliness as a subjective negative feeling associated with a perceived lack of a wider social network (social loneliness) or the absence of a specific desired companion (emotional loneliness)”* (p. 518).

There are good reasons to address issues of assessment, particularly in the context of bereavement. For example, not only is there need to reach consensus across different diagnostic sets of criteria for grief complications (Lenferink et al., 2021), but in the changed world of the COVID-19 pandemic, loneliness is likely to become an even greater issue, particularly among the bereaved. Predictions are that the lack of physical presence and support from family and friends will intensify feelings of loneliness even more than in non-pandemic times (Stroebe and Schut, 2020), with evidence beginning to confirm such prognoses (van Tilburg et al., 2020). Given these concerns, research needs to provide a body of sound information on the prevalence and intensity, correlates, and consequences associated with loneliness in bereavement. In order to do so effectively, accurate measurement is needed.

The purpose of this study was to examine the assessment of loneliness in bereavement; to summarize the current state of knowledge; to chart how loneliness has been operationalized and interpreted to date; and to suggest ways forward for future research. To do so, we reviewed the existing body of scientific literature available on this topic. More specifically, we sought to explore:

- Which measures/questionnaires have been used?
- How have they (differentially) assessed loneliness?

## Method

Information for the present review was drawn from published, empirical studies designed to examine loneliness among bereaved people that we considered in our systematic review on the prevalence, correlates, and intervention efficacy of loneliness in bereavement (Vedder et al., 2021). Measurement issues, such as widespread use of single-item measures and heterogeneity in validated scales, arose from the results of that review but were beyond its scope, suggesting the need for finer-grained examination of the assessment of loneliness.

As described in detail in Vedder et al. (2021), studies conducted before March 12th, 2020 were included. The studies were selected according to the guidelines of the Preferred Reporting Items for Systematic Reviews and Meta-Analyses statement (PRISMA; Moher et al., 2009). The search in Psychinfo, Web of Science, Scopus, Medline, and PubMed returned 8,119 articles, of which 5,600 duplicates were removed, leaving 2,519 (31%) articles for screening. Following title and abstract screening, 312 (12%) articles were retained, and after full-text screening there were 63 articles that conformed to the set criteria for inclusion, representing 51 independent datasets. In all, 16,558 bereaved persons participated in the studies; their mean weighted age was 65 years (*SD* = 14). There were almost equal numbers of cross-sectional (*N* = 30, 59%) and longitudinal (*N* = 25, 49%) studies, but very few (*N* = 5, 10%) investigating the efficacy of interventions. Note that this sum exceeds the number of 51 independent datasets, as some were used for more than one study, with different designs. For the present review, articles were scrutinized to extract information on the measures used in each of the studies. Information was compiled, listing different versions of established scales, their instructions, the particular questions asked in single- or few-item measures, and whether the latter were self-constructed or derived from established scales. Answer categories in terms of response options, and the frequency and/or intensity of experienced loneliness were listed.

## Results

An overview of the measures used in the reviewed studies is included in the supplementary table 1 of Vedder et al. (2021). Tables 1–4 below summarize this information according to the type of measure employed to examine loneliness in the bereavement studies. As can be seen, a variety of scales has been used for assessment. Surprisingly, only about half of the studies used a validated measure, including either the UCLA Loneliness Scale (UCLA-LS; Russell et al., 1978; Russell, 1996; Elphinstone, 2018) or the De Jong Gierveld Loneliness Scale (DJG-LS; De Jong Gierveld and Van Tilburg, 1999, 2010). Other validated scales used (never more than once among those found through our search system) were the modified New York University Loneliness Scale (Rubenstein and Shaver, 1982), Emotional and Social Loneliness Scale (Russell et al., 1984; Robinson et al., 2013) and Emotional/Social Loneliness Inventory (Vincenzi and Grabosky, 1987). We consider the frequently-used scales next, before turning to the use of single- or few-item measures.

**Table 1:** Versions of the UCLA Loneliness Scale (UCLA-LS) used to measure loneliness (after bereavement).

**UCLA-LS**	**# Items**	**Scale**	**Range**
Revised UCLA-LS or UCLA-LS Version 3 (Hansson et al., 1986; Murphy, 1986; Gfellner and Finlayson, 1988; Kovarsky, 1989; Byrne and Raphael, 1997; Henderson et al., 2004; Stein et al., 2009; Knowles and O'Connor, 2015; Yan and Bonanno, 2015; Spino et al., 2016; Cao et al., 2020)	20 items	never, rarely, sometimes, often/always	0–80
UCLA-LS-Short Form (Lund et al., 2010)	13 items	never, rarely, sometimes, always	13–52
UCLA-LS-8 (Lee, 2019)	8 items	strongly disagree (1)—strongly agree (5)	8–40
UCLA-LS-4 (Zettel and Rook, 2004)	4 items	agree a lot (1)—disagree a lot (5)	4–20
UCLA-LS-4 (Morgan et al., 1997)	4 items	never, rarely, sometimes, often	4–16
UCLA-LS-5 (Sun et al., 2012)	5 items	agree almost always (1) to almost never (5)	5–25
UCLA-LS-3 (Carr et al., 2018)	3 items	hardly ever, some of the time, or often	0–9

### Scales Measuring Loneliness in Bereavement

A third of the studies (*N* = 17, 33%) used a version of the UCLA-LS, a well-established measure of loneliness (see Russell, 1996, for portrayal of the questionnaire). The scale has shown dependably high internal consistency, with a coefficient alpha of >0.90 in college student populations and good retest reliability after 12 months (*r* = 0.73) (Cramer and Barry, 1999). Nearly all of the studies using a version of this scale were conducted in the U.S. (*N* = 15, 29%), the country of origin of the scale developers. The UCLA-LS was based on an early conceptualization of loneliness (Russell et al., 1978). The emphasis on uni-dimensionality is noteworthy (Russell, 1996, p. 30), contrasting with the emotional and social loneliness dimensionality of the DJG-LS (see below). Russell et al. (1980), see also Russell et al. (1984) further described a cognitive model of loneliness in which the perception is that “social relationships are “too few,” and people feel “lonely” (Russell et al., 1980, p. 472). Thus, consistent with what is usually understood as the nature of the experience, these researchers placed emphasis on the person's own subjective perception of loneliness. It is worth noting that intensity as well as frequency were incorporated (as reflected in the scale).

The second most-frequently used scale in bereavement studies is the De Jong Gierveld Loneliness Scale (*N* = 8, 16%) (DJG-LS; De Jong Gierveld and Van Tilburg, 1999, 2010). Internal consistency is reported to be good, particularly among older adult samples, with Cronbach's alpha coefficients ranging from 0.80 to 0.90 (De Jong Gierveld and Van Tilburg, 1999; for comparison of the UCLA-LS with the DJG-LS see Penning et al., 2014). Overall, authors have concluded the scale demonstrates reliability and validity (De Jong-Gierveld and Kamphuis, 1985; Pinquart and Sörensen, 2001a,b; Gierveld and Tilburg, 2006). Nearly all of the studies using this scale were conducted in The Netherlands (*N* = 6, 75%), again the country of origin of the scale developers. The DJG-LS followed a definition of loneliness as “the manner in which the person perceives, experiences, and evaluates his or her isolation and lack of communication with other people” (De Jong-Gierveld, 1987, p. 120). The DJG-LS therefore emphasizes some rather different components of loneliness (e.g., communication) compared with the UCLA-LS. However, quite similarly to the DJG-LS, loneliness is here considered to be a subjective negative feeling originating from perceived deficits in social relationships, indicating a lack of intimacy or support in relationships, one that reflects a person's social participation and isolation. The scale also adopted the developers' so-called “cognitive theoretical approach” (De Jong Gierveld and Van Tilburg, 1999), which emphasizes the discrepancy between what the person *desires* in terms of interpersonal affection and intimacy and what one *perceives they actually have*. Again, this is similar to the cognitive approach reflected in the UCLA-LS. Furthermore—and uniquely at the time the scale was developed—De Jong Gierveld and colleagues built on Weiss's (Weiss, 1973) constructs of social and emotional loneliness, as compared with the unidimensional measurement approach reflected in the UCLA-LS.

Turning to the actual use of these loneliness scales in bereavement research: Within the empirical studies examined, different versions of the UCLA-LS–including various shortened versions–have been utilized (see Table 1; adapted from Vedder et al., 2021). Instructions ask respondents to indicate how often each of the statements applies to them. Items are typically rated on a 4-point scale, from “never feeling this way” to “often feeling this way.” Inspection of these scales shows that the items do not include the actual word *loneliness* (we consider this strategy in Discussion), not even in the instructions, in order to reduce response bias. Instead, they cover aspects such as: lacking companionship, having no one to turn to, feeling isolated, feeling unhappy being so withdrawn. As such, they reach beyond the above-cited definition of loneliness (Valtorta and Hanratty, 2012), to include social isolation as well as related concepts that could function as moderators, mediators, or outcomes (e.g., the included item about unhappiness). Such extensions in the scope also represent a systematic difference compared to the single- or few-item measures of loneliness (discussed below). As Cramer and Barry (1999, p. 493) note, the UCLA-LS does not specify a time frame for respondents, so it remains “unclear whether a state or trait measure has been designed.” There appears to be no norm or reference information concerning UCLA-LS scores, limiting the extent to which scores from a particular study can be compared with those found for other populations or relevant sub-groups. Nor do there appear to be agreed-on points for designation of intensity, for example, to indicate absence of, mild, moderate or high levels of loneliness (Russell et al., 1978, 1980; Weeks et al., 1980). Given the number of different versions of the UCLA-LS that have been used by researchers, it is also difficult to compare results across studies.

As with the UCLA-LS, different versions of the DJG-LS have been employed in bereavement research, with most using the full 11-item scale, while some used the six-item version (see Table 2). Like the UCLA-LS, this scale does not include the word *loneliness* at all, covering such aspects as having no one to share problems with, experiencing a sense of emptiness, feeling rejected, and having no one to trust completely. Thus, the scope seems somewhat broader than the definitions provided by the authors, raising questions about content validity. Instructions were slightly more elaborate than those for the UCLA-LS, explaining that the statements were made by individuals who had previously shared their experience with the researchers, and asking respondents to indicate the extent to which they applied to their own situation, the way they currently felt. However, as with the UCLA-LS, no specific time frame was included. In contrast to the use of a four-point Likert scale for the UCLA-LS, answers for DJG-LS items are scored by circling the statements that applied to the respondent's situation. Notably, a cut-off point relating to severity was provided (De Jong Gierveld and Van Tilburg, 1999). Furthermore, the DJG-LS and its shorter versions enable overall loneliness (from the total scale) as well as emotional and social loneliness (from subscales) to be calculated, facilitating comparison across studies.

**Table 2:** Versions of the De Jong Gierveld Loneliness Scale (DJG-LS) used to measure loneliness (after bereavement).

**DJG-LS**	**# Items**	**Range**	**Cut-off**
DJG-LS (Stevens, 1995; Van Baarsen et al., 1999; De Groot et al., 2006; Onrust et al., 2010; Merz and De Jong Gierveld, 2016; Szabo et al., 2019)	11	0–11	0–2 not lonely; 3–8 moderately lonely; 9–11 strongly lonely;
DJG-LS Short scale (Spahni et al., 2015; Chow et al., 2019)	6	0–6	0–1 not lonely; 2–6 lonely

### Single-Item or Few-Item Measures to Assess Loneliness in Bereavement

More than a third of the studies used single- or few-item measures to assess loneliness (*N* = 19, 37%). Such items were used across more countries than either of the above validated scales: Half of the studies (*N* = 9, 50%) were conducted in various European countries, a lesser proportion was conducted in the U.S. (*N* = 7, 39%). The remainder were either conducted in China (*N* = 2, 11%) or internationally-administered (*N* = 1, 6%). A number of the short measures were derived from existing scales (*N* = 7, 14%; see Table 3). Instructions (not listed here) varied according to the overall focus of the existing scales from which they were derived, and/or the interests of the particular researcher who developed the short measure.

**Table 3:** Single items derived from validated measures to assess loneliness (after bereavement).

**References**	**Scale**	**Single-item measure**
Robinaugh et al. (2014) and Fried et al. (2015)	Center for Epidemiologic Studies Depression Scale	“I feel lonely”
D'Epinay et al. (2003)	Self-Assessing Depression Scale	“I feel rather isolated, rather lonely, even among friends”
Pan (2020)	Inventory of Complicated Grief	“I feel lonely a great deal of the time ever since. died”
Waldrop (2007)	Brief Symptom Inventory	“Feeling lonely,” “Lonely with people”
Xiang et al. (2016)	Psychosomatic Situation Scale	“Feeling lonely,” “Lonely with people”
Oechsle et al. (2020)	Distress Thermometer	Participants can answer “yes” or “no” to whether it was loneliness causing their distress.

Other studies—accounting for ~one-quarter of the total (*N* = 12, 24%)—used items that were self-constructed by the authors (see Table 4). A noteworthy difference between these single- or few-item measures and the two validated scales discussed above is that their items all refer explicitly to loneliness, with each of them also employing Likert-type scales. For example, Eckholdt et al. (2018) used a 2-item, 7-point scale of emotional loneliness: “*I feel lonely even when I am with other people”* and social loneliness: “*I have no really close friends.”* Savikko et al. (2006), used a 1-item, 3-point one: “*Do you suffer from loneliness?”* with answer categories from 1 = seldom or never, 2 = sometimes, 3 = often or always.

**Table 4:** Self-constructed items to measure loneliness (after bereavement).

**References**	**Single-item measure**
Abrahams (1972)	Help-request used and categorized in (1) lonely requires a listener and (2) lonely wants to meet people
Arling (1976)	Two items (1) “Do you have as much contact as you would like with a person that you feel close to- somebody that you can trust and confide in?” (2) “Do you find yourself feeling lonely quite often, sometimes or almost never?”
Atchley (1975)	Participants could answer if they feel lonely “Lots, Some, Hardly Ever or Not all”
Bahr and Harvey (1979)	Two items (1) from Bradburn (1969): “During the past week, how often did you feel very lonely or remote from other people?” (3-point scale). (2) item “Are you as involved in community life as you would like to be?” (yes/no).
Caserta and Lund (1996)	Single item (1 = not at all lonely, 7 = very lonely)
Grimby (1993)	No scale but three dimensions of grief reactions (as rated by psychologists). Under which “low mood” = dysphoria, loneliness, crying and pessimism.
Eckholdt et al. (2018)	Two items (7-point scale)—Emotional loneliness “I feel lonely even when I am with other people” and Social loneliness “I have no really close friends.”
Kivett (1978)	Single item “Do you find yourself feeling lonely quite often, sometimes, or almost never?” 3-point scale
Lichtenstein et al. (1996)	Loneliness: Single item “How often do you feel lonely?” 3-point scale “almost never or never” (0) to “almost always or always” (3).
Savikko et al. (2006)	Loneliness: 1 item “Do you suffer from loneliness?” 1 = seldom or never, 2 = sometimes and 3 = often or always
Stroebe et al. (1996)	Self-constructed measure of social (2 items) and emotional (2 items) loneliness (based on Weiss, 1982), yes/no answers
van der Houwen et al. (2010)	Emotional loneliness, 2 items: “I feel lonely even if I am with other people”; “I often feel lonely.” 7-point scale ranging from 1 (totally disagree) to 7 (totally agree).

## General Comments

Some general comments on scale use are called-for, to elaborate on certain emerging patterns that arose during the course of this review. We noted a number of differences between the various measurement instruments in terms of content, instructions, and answer categories. We also noted that there were different applications of measures across countries, with more international coverage in the use of single- or few-item measures than for either of the validated scales. Given these results, we explored other potential differences: We considered it plausible that single- or few-item measures might have been employed mostly in the years prior to the establishment of the validated scales; this, however, was not the case. More than half of the studies employing single- or few-item scales (*N* = 10, 56%) were conducted since the year 2000, well after both validated scales were developed. We also considered the possibility that researchers would choose to ask older participants fewer questions, to avoid burdening them with more items (or even to limit response burden for participants in general). This hypothesis was also not supported; the mean ages of participants in studies using the longer scales or short-item scales did not show any notable differences. We reflect on these results further in the Discussion section.

Overall, given the diversity of measurement options described above and in Tables 1–4, it is difficult to establish precise rates for prevalence or intensity of loneliness in bereavement, which is clearly relevant for clinical awareness and intervention planning (for further discussion: Vedder et al., 2021). From single-item measures, for example, we mainly learn about percentages of people who are lonely, i.e., how prevalent loneliness is. Prevalence and intensity could be investigated by using the UCLA-LS, but in the absence of any agreed-upon cut-off point, researchers are left to derive their own interpretations, or find other studies using comparable samples and measurement to derive their own conclusions. We also noted the wide variety of versions of measurement instruments that researchers have used. This could lead to different claims about the impact of loneliness in bereavement. For example, with the 6-item DJG-LS version, one can only infer “lonely” vs. “not lonely,” whereas with the full 11-item version, four grades of loneliness (not lonely, moderate lonely, severe lonely and very severe lonely) are identifiable. Finally, it is noteworthy that approximately half of the reviewed studies (*N* = 26, 51%) employed scales consisting of fewer than 10 items. This might reflect the necessity for researchers to tailor their study design to the specific characteristics of their participants, and/or to reduce participant burden.

## Discussion

Our review of studies of loneliness in bereavement revealed diversity with regard to many central features relating to its assessment. Several of these are noteworthy: There is an abundance of different (versions of) scales and items concerning loneliness; a wide variety of instructions and answer categories are used; there is limited information about how to interpret scores, intensity or cut-off points for clinically-relevant levels of loneliness. Furthermore, the use of different scales and/or versions results in lack of comparability between different studies, and across different populations or sub-groups. Examination of the full body of evidence also shows that, depending on which measure is employed, one might draw different conclusions about prevalence and/or intensity of loneliness. These problems associated with the assessment of loneliness after bereavement add to other methodological limitations of the empirical studies, discussed elsewhere (see Vedder et al., 2021). For instance, the majority of these studies lack non-bereaved control groups and longitudinal assessments.

A series of critical questions remain: What can we learn from the way loneliness has so far been measured in empirical studies on bereavement? How can researchers build on existing information to improve assessment and, consequently, contribute to knowledge in general about the role of loneliness in bereavement? Several points relating to the assessment of loneliness must be taken into account for future research:

### Definition of Loneliness

First, it would be advisable to come to a clearer agreement on the definition of what loneliness is—and is not. The widely-used definition by Valtorta and Hanratty (2012) reflects basic components identified in both theorizing and empirical research. Details of the measures included in our review seem in line with this general definition: They focus on subjective negative feelings and some enable distinction of social from emotional loneliness. The latter distinction seems critical when considering loneliness after bereavement, in line with theoretical formulations. In bereavement, one has lost an attachment figure (Weiss, 1973; Bowlby, 1980) a person to whom one was closely bonded, suggesting the centrality of emotional rather than social loneliness (although social loneliness may be particularly felt among older bereaved persons as their networks decrease). Indeed, there is some empirical support for this supposition (Stroebe et al., 1996; Guiaux, 2010). De Jong Gierveld et al. (2006), provide further discussion of the need for distinction of social from emotional loneliness.

Thus, it may be helpful to use the description by Valtorta and Hanratty (2012) as a working definition for further research; others have endorsed this strategy for research on loneliness (Fakoya et al., 2020). However, openness to alternatives may also be called for. Cramer and Barry (1999) have provided a valuable evaluation and comparison of the various self-rating scales (including the UCLA-LS and DJG-LS). They recommend use of the Social and Emotional Loneliness Scale for Adults (SELSA; DiTommaso and Spinner, 1993, 1997). They add the factor “family loneliness,” a feature that is well in-line with our argument that emotional loneliness is of particular interest when studying bereavement. Furthermore, Bandari et al. (2019) are currently reviewing definitions of loneliness and, on the basis of the accumulated knowledge, are planning to propose a new definition that may better reflect the scientific construction of what loneliness incorporates.

### Avoidance of Conceptual Overlap

There is need to avoid conceptual overlap with related constructs such as social isolation and social support. Yanguas et al. (2018) state that “…various researchers have referred to “loneliness” and “social isolation” indistinctly. Others, however, find both terms very different from each other. Making accurate evaluations depends on a clear definition of the concept of loneliness, with special awareness of its multidimensionality and its differences with respect to related concepts (social isolations or a lack of social support)” (p. 302–303). Our review showed that some measures cover the latter dimensions in their items, while others do not. Researchers may want to adapt the content of the scales to conform to the narrower definition of loneliness, in order to better distinguish it from related—yet distinct—constructs.

### Selection of an Appropriate Measurement Instrument

Our results showed that two scales, the UCLA-LS and the DJG-LS, have each been quite widely adopted. A comparative examination of the UCLA-LS with the DJG-LS concluded that the latter has better utility, at least for use among middle-aged and older persons (Penning et al., 2014). However, some of the DJG-LS items border on different domains (e.g., feeling rejected or not having people one can trust). Such items might best be deleted, and/or items from the UCLA-LS that are complementary to those on the DJG-LS added (requiring new validation). The DJG-LS has the advantage of enabling examination of emotional loneliness and social loneliness as well as overall loneliness.

Deciding whether to assess loneliness using a validated instrument or a single/few-item measure requires careful weighing of pros and cons. There is much to support the use of an established scale. Multiple-item usage increases reliability and validity; errors and the specificity that are inherent in single items are averaged out (Bowling, 2005). For such reasons, instruments with carefully selected items are *almost* always considered superior to single item measures in scientific research. However, the qualification *almost* may be particularly appropriate for assessing loneliness in bereavement. For instance, the average age of participants in the reviewed studies was 65 years, suggesting a wide interest in loneliness among older bereaved persons, for some of whom research participation may be more challenging than for younger persons. When study participants are overwhelmed by the length of an interview or the cognitive demand of responding to multiple scale items, using multi-item scale measures may not be the better choice: One well-answered item clearly has superiority over a number of unanswered or incorrectly answered ones. In addition to feasibility, when the goal is to assess the general feeling of loneliness rather than distinguish different dimensions of it, one or a few items may be sufficient. The fact that approximately half of the reviewed studies used ten items or fewer may reflect this dilemma. For some purposes, few items may be able to capture loneliness as well as the score on a longer loneliness scale. Finally, the choice depends on the broader aims of the study (e.g., symptomatology in general vs. loneliness in particular).

In summary: Choice of one of the validated scales or a single/few-item measure likely reflects the distinct aims and scopes of the studies, as well as the targeted participants. Indeed, different types of assessment may be called for under specific circumstances. There is no one-size-fits-all solution to the measurement of loneliness in bereavement.

### On the Use of an Indirect or Direct Measure

Another issue that the scope of the reviewed studies raises is whether one should ask directly about being lonely, or whether some proxy is preferable^1^. On the one hand, one might argue that people in general—and perhaps bereaved people in particular—are likely to know whether or not they feel lonely (and/or how intensely); thus, there is no clear detriment to asking directly about loneliness. However, on the other hand there is risk of social desirability bias, and demand characteristics or perceived expectations may prevail (e.g., a societal norm may prevent someone from admitting to being lonely). In this case, indirect questions may garner more accurate information about loneliness from respondents than direct questions. An example of an indirect item (from DJG-LS) is: “I miss having a really close friend.”

Apart from the content of the items, we noted that response categories used in the scales and single/few-item measures varied among the reviewed studies, ranging from simple “yes”/“no” options to Likert scale usage and to further sub-categorization (e.g., collapsing a larger range of scores into three categories, such as “not lonely,” “moderately lonely” and “strongly lonely”). Again, there would be advantages to coming to agreements where possible on this point, to enable comparison of results across studies. More research is needed to evaluate the pros and cons of different response option formats.

### Comparing Measures

To work toward selection of the most appropriate, reliable, and valid measurement of loneliness for any particular investigation, one strategy would be to compare results on the existing instruments with single- or few-item measures. None of the studies we reviewed used both single/few-item measures in addition to validated scales.^2^. For practical purposes, if a short measure is indicated, the best-loading items of emotional and social loneliness could be selected. However, this may result in a further expansion of loneliness measures. On the other hand, a validated pool of items could be established from which clinicians and researchers may select. In addition, we refer above to the review of loneliness scales by Cramer and Barry (1999), a valuable source for considering the relative merits of different assessment instruments.^3^

It would also be useful to examine the benefits of the self-constructed items, that is, ones created by the investigators themselves, based on their understanding of “loneliness” (Table 4) compared with those selected from the established, more generic scales (Table 3), such as the Center for Epidemiologic Studies Depression Scale (CES-D) or Symptom Checklist-90 (SCL-90), ones which have also already been used in bereavement research. This would help to improve standardization and optimization of single/few-item measurement of loneliness across studies, allowing for comparison across study contexts and (sub)populations.

### Limitations

Additional limitations to the data set and our review process need to be mentioned. First, our criteria limited the scope of investigation: notably, they restricted the review to adults, excluding bereaved children; only pre-COVID-19 studies were covered; only English language articles were included. Second, our chosen focus was on bereavement and not on other situations or diagnostic categories; comparisons across such domains would also be enlightening (for review of relationships between loneliness and psychiatric as well as physical disorders: Mushtaq et al., 2014). As a result of these selection criteria, the number of participants in the studies reviewed ranged from 30 to 2,018, and most participants were bereaved through loss of their spouse. The average age of sample participants, as noted above, was 65, limiting our ability to examine loneliness in bereavement across different age groups. However, there was no preferred use of specific assessments with regards to study-size or target group. Finally, regarding the available studies: to the best of our knowledge, no scientific investigation has yet been undertaken to compare the relative usefulness of the different measures to assess loneliness in bereavement. For recommendations to be made, such systematic, comparative investigations of the measures are needed.

## Conclusion

The variety of assessment tools used in the reviewed studies makes it difficult to come to conclusions about the prevalence, intensity, and influence of loneliness in bereavement. Taking the effect of loneliness on quality of life and health seriously, selection of the most reliable, valid, and feasible measure(s) is needed to move the field forward (e.g., toward finer-grained examination of variables/mechanisms influencing loneliness or for planning psychotherapeutic intervention). While our original aim in reviewing the assessment of loneliness was to determine which (possibly adapted) instrument might be chosen for universal, common use, closer examination of the instruments suggested that “one size does not fit all”; different measures are needed for different purposes, and for application among different subgroups. Thus, our review should be taken as a source of presently available information, as well as a discussion of the issues currently facing researches of loneliness in bereavement. Additional research and reflection is needed to work toward more concrete guidelines to establish which instrument to use in what context; our inventorization is a suggested step in this direction.

## Author Contributions

AV and MS drafted the manuscript. PB and KB discussed and commented on the text, providing suggestions for improvement. All authors were involved the conceptualization and discussion as well as critical revision of the manuscript and approved the final version of the article.

## Conflict of Interest

The authors declare that the research was conducted in the absence of any commercial or financial relationships that could be construed as a potential conflict of interest.

## Publisher's Note

All claims expressed in this article are solely those of the authors and do not necessarily represent those of their affiliated organizations, or those of the publisher, the editors and the reviewers. Any product that may be evaluated in this article, or claim that may be made by its manufacturer, is not guaranteed or endorsed by the publisher.

## Footnotes

^1^Useful discussions relating to this issue have appeared in the literature (Cramer Cramer, K.M., and Barry, J.E. (1999). Conceptualizations and measures of loneliness: A comparison of subscales. *Personality and Individual differences* 27(3), 491-502. doi: https://doi.org/10.1016/S0191-88699800257-8.) (e.g., ibid., Victor et al., 2005). Measuring loneliness in later life: a comparison of differing measures. *Reviews in Clinical Gerontology* 15(1), 63. doi: https://doi.org/10.1017/S0959259805001723, van Tilburg et al. (2020). Loneliness and Mental Health During the COVID-19 Pandemic: A Study Among Dutch Older Adults. *The Journals of Gerontology: Series B*, gbaa111,. doi: https://doi.org/10.1093/geronb/gbaa111.

^2^For further information from a recent non-bereavement-specific study of loneliness which did so, see von Soest et al. (2020). Development of loneliness in midlife and old age: Its nature and correlates. *Journal of Personality and Social Psychology* 118(2), 388-406. doi: http://dx.doi.org/10.1037/pspp0000219, ibid.

^3^For a review on loneliness measures in children and adolescence, see Cole et al. (2021). A Systematic Review of the Development and Psychometric Properties of Loneliness Measures for Children and Adolescents. *International Journal of Environmental Research and Public Health* 18(6), 3285. doi: https://doi.org/10.3390/ijerph18063285.
